# Virucidal Potential of Citric Acid, Lemon Juice and Lemon Essential Oil Against Influenza D Virus

**DOI:** 10.3390/pathogens15101034

**Published:** 2026-10-01

**Authors:** Alessandro Falsini, Aurora Fiori, Simone Cataldini, Francesca Finetti, Mariangela Gentile, Raffaello Nardin, Gabriella Tamasi, Claudio Rossi, Pietro Lupetti, Michele Camero, Emanuele Montomoli, Claudia Maria Trombetta

**Affiliations:** 1 Department of Molecular and Developmental Medicine, University of Siena, 53100 Siena, Italy; alessandro.falsini2@unisi.it (A.F.); aurora.fiori@unisi.it (A.F.); montomoli@unisi.it (E.M.); 2 Department of Biotechnology Chemistry and Pharmacy, University of Siena, 53100 Siena, Italy; simone.cataldini2@unisi.it (S.C.); raffaello.nardin2@unisi.it (R.N.); gabriella.tamasi@unisi.it (G.T.); claudio.rossi@unisi.it (C.R.); 3 Department of Life Science, University of Siena, 53100 Siena, Italy; finetti4@unisi.it (F.F.); mariangela.gentile@unisi.it (M.G.); pietro.lupetti@unisi.it (P.L.); 4 Department of Veterinary Medicine, University of Bari, 70010 Valenzano, Italy; michele.camero@uniba.it; 5 VisMederi srl, 53100 Siena, Italy; 6 VaepiX, Joint Research Laboratory, University of Siena, 53100 Siena, Italy

**Keywords:** influenza D virus, lemon essential oil, lemon juice, natural antiviral

## Abstract

Influenza D virus (IDV) is an emerging pathogen with zoonotic potential. Epidemiological and serological evidence suggests that cattle serve as the natural reservoir, with periodic spillover events to other animal hosts. This study evaluates the in vitro activity of lemon juice (LJ) and lemon essential oil (LEO) against two IDV lineages (D/660 and D/OK). Compound cytotoxicity was evaluated on MDCK cells by measuring cell viability with a fluorescein diacetate assay. Viral suspensions were pre-treated with the compounds, and the viral titers were determined by TCID_50_. Immunofluorescence analysis was performed to evaluate viral nucleoprotein expression. Then, the potential morphological changes in treated viral particles were investigated with transmission electron microscopy. Both LJ and LEO showed significant dose- and time-dependent antiviral effects, leading to a rapid reduction in viral titers at higher concentrations and at non-cytotoxic levels. Moreover, at the highest non-cytotoxic dilutions, prolonged exposure (30–60 min) resulted in a reduction of approximately 1–1.5 log_10_ TCID_50_/mL in viral titers. Moreover, treatments reduced viral nucleoprotein expression and were associated with morphological alterations of viral particles observed by transmission electron microscopy, providing supportive evidence for a possible direct effect on virion integrity. These findings suggest that lemon-derived compounds possess promising properties against IDV and may represent potential natural agents for disinfectant or virucidal applications.

## 1. Introduction

Influenza viruses, belonging to the *Orthomyxoviridae* family, represent one of the most significant respiratory pathogens affecting both humans and animals worldwide. Currently available antiviral drugs, such as neuraminidase inhibitors (e.g., oseltamivir) and M2 ion channel blockers, face increasing viral resistance and limited effectiveness [1].

Influenza D virus (IDV) is a recently identified member of the *Orthomyxoviridae* family [2]. Multiple epidemiological and serological studies support the hypothesis that cattle serve as primary amplification hosts and natural reservoirs of IDV, with periodic spillover events to other animal hosts [3,4,5]. More recently, serological evidence has also reported IDV in cats and dogs in China, Italy and Ukraine [6,7]. Although initially considered incapable of infecting humans, seropositivity observed not only among cattle and swine workers but also in the general population suggests that zoonotic infections may already be occurring [8,9,10,11]. Experimental studies using both standard human lung epithelial cell lines and primary well-differentiated human airway epithelial cultures have demonstrated that IDV can efficiently infect human respiratory epithelial cells, achieving replication titers comparable to those of influenza A viruses [12]. Moreover, IDV has been detected in bioaerosol samples collected from airport environments [13] and hospital emergency rooms [14], suggesting possible environmental circulation and human exposure beyond direct occupational contact with infected animals. Taken together, the broad host range of IDV, its ability to infect human respiratory tissues, and its detection in environments with high human activity highlight the need for further investigation of this emerging virus and the identification of novel antiviral strategies that may contribute to controlling IDV circulation.

One distinctive feature of IDV compared with other influenza viruses is its remarkable stability under conditions of temperature and acidity variation [15]. For instance, influenza A viruses can be readily inactivated by various reagents, including sodium hypochlorite, ethanol, aldehydes, and common household disinfectants. In contrast, IDV has been shown to be considerably less sensitive to these agents, likely due to its intrinsic structural stability [16]. IDV also replicates efficiently in cell cultures at 37 °C, a temperature consistent with that of the human lung environment [17]. Moreover, the virus retains infectivity even after exposure to 53 °C for 2 h, maintaining approximately 40% of its viral titer [18]. Similarly, IDV preserves a high level of infectivity when exposed to acidic conditions, retaining about 80% of its infectious titer after treatment at pH 3.0 for 30 min, whereas influenza A, B, and C viruses show a marked reduction in infectivity under the same conditions [18].

The search for alternative or complementary antiviral strategies derived from natural products has gained increasing attention in recent years. Plant essential oils (EOs) and their active compounds exhibit a wide range of biological activities, including antibacterial, antifungal, and antiviral effects [19]. Among these, products derived from *Citrus limon* contain several bioactive molecules such as citric acid, vitamin C and flavonoids in the juice, as well as limonene, β-pinene, and γ-terpinene in the EO [20]. These compounds have been demonstrated to have potential antiviral properties [19]. For instance, both lemon juice (LJ) and lemon LEO have been reported to have virucidal activity against feline calicivirus, a surrogate for norovirus. In cell culture assays, significant reductions in viral titers were observed following prolonged exposure, suggesting disruption of viral particles or inhibition of viral infectivity at higher concentrations (e.g., up to ~1.25 log_10_ TCID_50_ reduction at 3020 µg/mL after 8 h). Consistently, lemon-derived products have been shown to significantly reduce viral titers across different exposure times [21]. Furthermore, further studies demonstrated the virucidal efficacy of LJ against feline calicivirus and caprine alphaherpesvirus-1, the latter used as a homologous animal model for human herpes simplex virus type 2 [22,23]. These findings suggest that both acidic pH and bioactive constituents may play key roles in mediating antiviral activity [23]. Additionally, LEO applied to contaminated soft fruits significantly reduced hepatitis A virus infectivity in vitro, supporting its potential application in antiviral sanitation strategies in food systems [24]. Mechanistic reviews of EOs suggest that volatile compounds present in citrus may interfere with viral envelope or capsid integrity and inhibit viral attachment. However, current evidence remains mostly preliminary and is often limited to surrogate models or in vitro experimental systems rather than demonstrating clinical antiviral efficacy in humans [25].

Taken together, these findings suggest that lemon-derived products, and plant EOs more broadly, may exert antiviral effects against different types of viruses through multiple mechanisms, including direct virucidal activity, inhibition of viral entry or replication, and modulation of host responses.

Although the antiviral activity of lemon-derived products has been investigated against several viruses, their efficacy against influenza viruses remains less explored, and no studies have evaluated their activity against IDV [26]. The selection of LJ and LEO in the present study was based on their complementary chemical composition. LJ is rich in organic acids, vitamin C and flavonoids, whereas LEO contains monoterpenes such as limonene, β-pinene and γ-terpinene, compounds that have been associated with virucidal activity against both enveloped and non-enveloped viruses through mechanisms including disruption of viral particles and interference with virus attachment or entry.

Given the increasing evidence of IDV circulation among different animal species and the remarkable environmental stability of IDV, including its resistance to acidic pH, evaluating whether these natural products retain antiviral activity against this emerging virus represents an important and previously unexplored research question. Indeed, as pointed out before, unlike influenza A, B, and C viruses, IDV exhibits remarkable environmental stability. Experimental studies by Yu et al. [18] have demonstrated that IDV retains substantial infectivity under conditions that efficiently inactivate the other influenza virus types, including exposure to acidic pH. However, experimental data on the susceptibility of IDV to conventional chemical disinfectants remain limited, emphasizing the need to evaluate candidate virucidal compounds under controlled experimental conditions.

Considering this, the present study aims to evaluate the antiviral potential of LJ and LEO against IDV.

## 2. Materials and Methods

### 2.1. Compounds

#### 2.1.1. Citric Acid

A 0.24 M citric acid solution (Sigma-Aldrich, Saint Louis, MO, USA) was prepared. In particular, pure anhydrous citric acid was dissolved in deionized water, without adding sodium citrate. The pH of the resulting 0.24 M citric acid stock solution was directly measured and confirmed to be 2.0 using a calibrated pH meter. The stock solution was subsequently diluted to obtain the working concentrations used in the virucidal assays. The initial citric acid stock solution was prepared at a concentration of 0.24 M. The working concentration obtained from the 1:2000 dilution corresponded to 0.12 mM (23 µg/mL). The higher concentrations,1:100 and 1:1000 dilutions, correspond to 2.4 mM (approximately 460 µg/mL) and 0.24 mM (approximately 46 µg/mL), respectively.

In the figures, CA treatments are reported according to the dilution factors used during sample preparation, consistently with the presentation of LJ and LEO, whereas the corresponding molar concentrations are provided in the figure legends.

#### 2.1.2. Lemon Juice

LJ was obtained by manually peeling fresh lemons of the *Citrus limon* “Florentina” variety, also known as *Citrus limon* “quattro stagioni,” which is available year-round throughout Italy. The peeled fruits (pulp only, without the peel) were then cold-pressed to obtain the juice at room temperature. The LJ was centrifuged and subsequently filtered through 0.22 μm polyvinylidene difluoride filters (Corning, Corning, NY, USA). The pH of the resulting juice was measured using a pH meter and was found to be 2.0.

#### 2.1.3. Lemon Essential Oil Production

**Figure 1:**
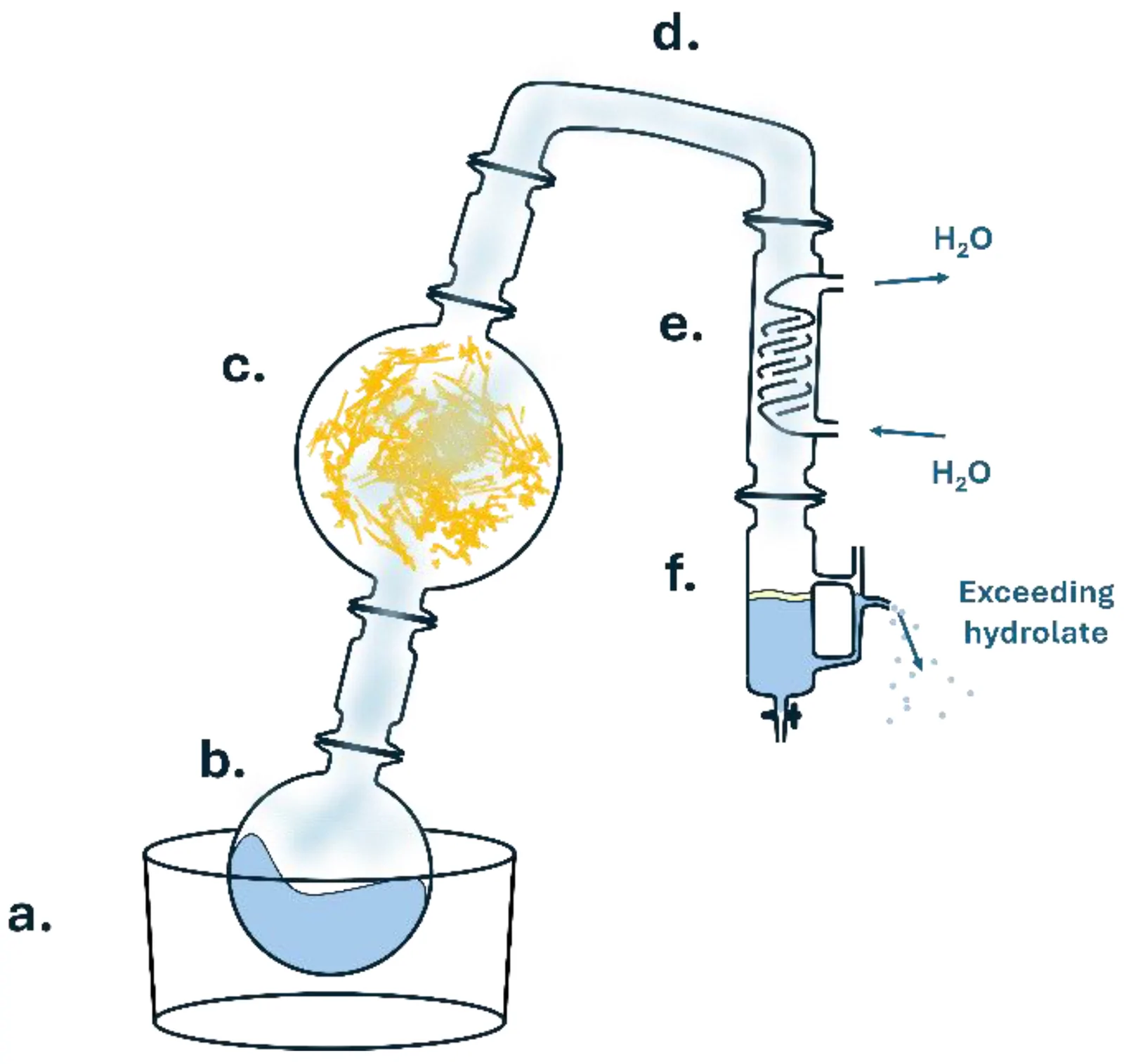
Schematic depiction of the steam-distillation setup followed. (a) Heating mantle; (b) boiling-water flask; (c) distillation head containing lemon peels; (d) bridging-glass connector; (e) Graham condenser; (f) collection flask.

Lemon peels were obtained from fresh lemons (*Citrus limon*) and subjected to steam distillation. The distillation setup utilized is schematized in Figure 1. All the peels were manually separated from the pulp, cut into slices, and used fresh. Briefly, water was allowed to boil in a 2 L round-bottom flask (b) via a heating mantle (a), while the lemon peels were compressed within the distillation head (c). The vapors traveled via a glass connector (d) to the Graham condenser (e), thereby recondensing within the collection flask (f), allowing, in turn, the physical separation of the LEO component as a pale yellow upper layer, due to its lower density with respect to the collected water. After 5 h, the LEO layer was collected and stored in glass vials at 4 °C. Out of the 258 g of initial lemon peels, 5.4 g of EO were obtained. LEO was dissolved in 1% dimethyl sulfoxide (DMSO) before use.

### 2.2. GC-MS Analysis of Lemon Essential Oil

LEO was characterized by means of gas chromatography–mass spectrometry (GC-MS) using an Agilent 8890 GC (Agilent, Santa Clara, CA, USA) system coupled to an Agilent 7000E GC/TQ (Agilent, Santa Clara, CA, USA) mass system. The sample was introduced via autosampler PAS RSI 85 Series 2 (Agilent, Santa Clara, CA, USA) using a split injection (100:1) with an injection volume of 1.0 µL. The injector operated at a temperature of 250 °C and was fitted with a deactivated split liner (straight, with glass wool); (Agilent, Santa Clara, CA, USA). C8-C20 *n*-alkane mix standard (Merck KGaA, Darmstadt, Germany) and EOs were both diluted in MS-grade *n*-hexane (Merck KGaA, Darmstadt, Germany) prior to injection (1:200 dilution factor). The sample was analyzed in triplicate.

Chromatographic separation was achieved using two identical HP-5MS UI capillary columns (Agilent Technologies; 15 m × 0.25 mm i.d., 0.25 µm film thickness) connected in series, using a PSD system able to compensate for the loss of pressure in the second half of the column, resulting in a total column length of 30 m. Helium was used as the carrier gas with a calculated flow rate of 1.0 mL/min in the first column and 1.2 mL/min in the second. Adequate chromatographic separation was achieved with the following temperature ramp: 35 °C held for 5 min followed by a 10 °C/min ramp up to 250 °C. A final clean-up step (300 °C; held for 1 min) followed each run, bringing the total run time to 27.5 min. Mass spectrometry detection was performed in electron ionization mode with a source temperature of 230 °C and an electron energy of 70 eV.

Identification of the compounds was done by comparing the mass fragmentation spectra and linear retention indices calculated relative to a C8–C20 *n*-alkane series with those found in the literature and the NIST23 library. A minimum relative area percentage (0.1% with respect to the highest peak) was used as a preliminary filter to remove minor compounds. Identification (minimum match factor, 70%) and estimation of area and area percentage were done using MassHunter Workstation Unknowns Analysis software (version 12.1 Build 12.1.938.3© Agilent Technologies, Inc. 2006–2023).

### 2.3. Influenza Viruses

Influenza D/bovine/Oklahoma/660/2013 virus (D/660 lineage, here referred to as D/660), kindly provided by Prof. Feng Li, University of Kentucky, and influenza D/swine/Italy/199724-3/2015 virus (Ref-SKU: 026V-04373) (D/OK lineage, here referred to as D/OK), obtained from the European Virus Archive (Emerging Viral Diseases Medical Faculty, 27 boulevard Jean Moulin, Marseille, France), were propagated in MDCK cells.

### 2.4. Viral Titration and Titer Determination

MDCK cells, at a density of 1.5 × 10^5^ cells/mL, were pre-incubated in 96-well plates for 4 h to allow cell attachment, in Eagle’s Minimum Essential Medium (EMEM) supplemented with 0.5% FBS, 1% penicillin/streptomycin and 1% L-glutamine (Euroclone, Pero, MI, Italy). The IDV lineages D/660 and D/OK were exposed to cytotoxic and non-cytotoxic concentrations of CA, LJ, and LEO for different times (1, 2, 5, and 10 min or 5, 10, 30 and 60 min). After exposure to the tested compounds for the indicated times, 10-fold serial dilutions of each virus–compound mixture were prepared, and 100 μL of each dilution point was transferred onto MDCK cells. Each dilution was tested in 8 replicate wells. The plates were then incubated for four days at 37 °C in a humidified atmosphere containing 5% CO_2_ and read for hemagglutination (HA) activity in the supernatant. For HA reading, 0.35% turkey red blood cells were incubated with the supernatant for 30 min. The virus titer, expressed as log10 TCID50/mL and calculated with the Spearman–Kärber method, was ≅5.5 log10 TCID50/mL for both D/660 and D/OK.

### 2.5. Virucidal Activity Assay Citric Acid

The virucidal activity of CA against IDV was evaluated by pre-treating the virus with different dilutions of the compound. We tested both the maximum non-cytotoxic dilution (1:2000, as determined in Section 2.8) and dilutions above the host cell cytotoxicity threshold (1:100 and 1:1000) to evaluate potential rapid virucidal effects. Briefly, 100 μL of each virus (D/660 and D/OK) were mixed with 1 mL of CA at the non-cytotoxic dilution and incubated at room temperature for 5, 10, 30, and 60 min. For the higher concentrations (1:1000 and 1:100 dilution factors), shorter incubation times of 1, 2, 5, and 10 min were applied. Following incubation, the mixtures were immediately subjected to serial 10-fold dilutions in EMEM to stop the virucidal action of CA before inoculation onto MDCK cells. EMEM contains sodium bicarbonate, which acts as a powerful buffer. We verified that, at the first dilution (10^−1^), the pH of the mixture was restored to the physiological range of 7.2–7.4, immediately neutralizing any residual virucidal activity of CA and preventing any pH-mediated cytotoxicity on the cell monolayer. Then, the virus–CA mixtures, along with untreated infected cells as the viral control, were subjected to viral titration. For each experimental condition, a matched untreated viral control was prepared by mixing 100 µL of the corresponding virus suspension with 1 mL of EMEM without the addition of the tested compound. The control samples were incubated at room temperature for the same exposure times as the corresponding treated samples and were subsequently subjected to the same serial 10-fold dilution procedure and titrated in parallel under identical conditions. All experiments were performed in triplicate.

### 2.6. Virucidal Activity Assay Lemon Juice

The virucidal activity of LJ against IDV was evaluated by pre-treating the virus with different dilutions of the compound. We tested both the maximum non-cytotoxic dilution (1:2000, as determined in Section 2.8) and dilutions above the host cell cytotoxicity threshold (1:100 and 1:1000) to evaluate potential rapid virucidal effects. Briefly, 100 μL of each virus (D/660 and D/OK) were mixed with 1 mL of LJ at the non-cytotoxic dilution and incubated at room temperature for 5, 10, 30, and 60 min. For the higher concentrations (1:1000 and 1:100 dilution factors), incubation times of 1, 2, 5, and 10 min were used. Following incubation, the mixtures were immediately subjected to serial 10-fold dilutions in EMEM. As before, we verified that at the first dilution (10^−1^) the pH of the mixture was restored to a physiological range of 7.2–7.4, immediately neutralizing any residual virucidal activity of LJ and preventing any pH-mediated cytotoxicity on the cell monolayer. Then, the virus–LJ mixtures, along with untreated infected cells serving as viral control, were subjected to viral titration. For each experimental condition, a matched untreated viral control was prepared by mixing 100 µL of the corresponding virus suspension with 1 mL of EMEM without the addition of the tested compound. The control samples were incubated at room temperature for the same exposure times as the corresponding treated samples and were subsequently subjected to the same serial 10-fold dilution procedure and titrated in parallel under identical conditions. All experiments were performed in triplicate.

### 2.7. Virucidal Activity Assay of Lemon Essential Oil

The virucidal activity of LEO against IDV was evaluated by pre-treating the virus with different dilutions of the compound. We tested both the maximum non-cytotoxic dilution (1:10,000, as determined in Section 2.8) and dilutions above the host cell cytotoxicity threshold (1:100 and 1:1000) to evaluate potential rapid virucidal effects. Briefly, 100 μL of each virus (D/660 and D/OK) were mixed with 1 mL of LEO at the non-cytotoxic dilution and incubated at room temperature for 5, 10, 30, and 60 min. For the higher concentrations (1:1000 and 1:100 dilution factors), incubation times of 1, 2, 5, and 10 min were used. Following incubation, the mixtures were immediately subjected to serial 10-fold dilutions in EMEM to stop the virucidal action of LEO before inoculation onto MDCK cells, thereby substantially reducing the concentration of LEO carried over into the cell cultures. Then, we verified that at the first dilution (10^−1^), the dilution of the mixture was restored to a physiological concentration, with an effect on the virucidal activity of LEO, preventing any cytotoxicity to the cell monolayer. However, since a dedicated neutralization-recovery control was not performed, residual LEO-mediated virucidal activity during the titration procedure cannot be completely excluded. Then, the virus–LEO mixtures, along with untreated infected cells serving as a viral control, were subjected to viral titration. For each experimental condition, a matched untreated viral control was prepared by mixing 100 µL of the corresponding virus suspension with 1 mL of EMEM without the addition of the tested compound. The control samples were incubated at room temperature for the same exposure times as the corresponding treated samples and were subsequently subjected to the same serial 10-fold dilution procedure and titrated in parallel under identical conditions. A DMSO vehicle control was included in parallel. Since LEO was dissolved in 1% DMSO, the final DMSO concentration in the virus–LEO mixture was approximately 0.91%. Vehicle-control samples were incubated and titrated in parallel with untreated and LEO-treated samples under identical conditions. All experiments were performed in triplicate.

### 2.8. Cell Viability Assay

Monolayers of MDCK cells were seeded in 96-well plates and maintained at 37 °C in a humidified incubator with 5% CO_2_. The cells were incubated with different dilutions of the tested compounds for 24, 48, 72, and 96 h. Serial dilutions ranging from 1:200 to 1:200,000 (1:200, 1:2000, 1:10,000, 1:20,000, and 1:200,000) were prepared to assess the maximum non-cytotoxic concentration. Particularly, at 1:200 dilution, the buffering capacity of the EMEM was not sufficient to buffer the pH of CA, resulting in a final pH of approximately 4.5 to 5.0. Conversely, at 1:2000 and above, the sodium bicarbonate buffering system of EMEM successfully neutralized the mixture, restoring and maintaining the pH at a physiological range of 7.2–7.4, with no negative effect on cell survival.

Cell viability was evaluated using the fluorescein diacetate assay (Merck Life Science S.r.l., Darmstadt, Germany). Fluorescein diacetate was prepared as a 1 mg/mL stock solution in acetone and diluted 1:200 prior to use. Subsequently, 100 µL of the working solution was added to each well, and the cells were incubated for 30 min at 37 °C in the dark. Fluorescence was measured using a microplate reader (FLUOstar Optima, BMG Labtech, Durham, NC, USA) with excitation and emission wavelengths of 494 nm and 518 nm, respectively.

### 2.9. Immunofluorescence Analysis

For immunofluorescence analysis, 25,000 cells were seeded into 24-well plates with 10 mm diameter coverslips for each condition. D/660 and D/OK viruses were pre-incubated with the test compounds for 1 h and then added to the cells for 96 h. Cells were washed with PBS and fixed and permeabilized with methanol at −20 °C. The primary antibody detecting FLU D NP (Hölzel Diagnostika Handels, GmbH, Cologne, Germany) was diluted 1:250 in PBS with 2% BSA and incubated overnight at 4 °C in a humidified chamber. The next day, after 2 washing steps with PBS containing 0.2% BSA, an anti-rabbit 550-conjugated secondary antibody (Thermo Fisher Scientific, Cleveland, OH, USA) was added at a 1:300 dilution, and the samples were incubated for 1 h at room temperature. Wells were washed with PBS 0.2% BSA, and coverslips were mounted onto glass microscope slides. Images were acquired using a 63× objective on a ZEISS LSM700 confocal microscope (Oberkochen, Germany) and analyzed with ImageJ software (https://imagej.net/ij/docs/index.html (accessed on 1 June 2026)). Fluorescence intensity was expressed as Corrected Total Cell Fluorescence using the formula:

(CTCF) = integrated density − (area of selected cell × mean fluorescence of background readings).

Precisely, CTCF was selected as a quantitative parameter to estimate intracellular nucleoprotein (NP) accumulation, as variations in fluorescence intensity reflect changes in the amount of viral NP expressed within infected cells. At least 25 cells per condition were analyzed in each independent experiment.

### 2.10. Transmission Electron Microscopy

D/660 and D/OK viruses were incubated with the test compounds for 1 h prior to sample preparation. For each sample, 3 µL was loaded onto formvar-coated 300 mesh Cu grids for 5 min. The excess was removed by blotting with filter paper. Subsequently, 3 µL of 1% uranyl acetate in distilled water or 1.5% ammonium molybdate in distilled water was added to the grids for 30 s. The grids were then air-dried and exposed to formaldehyde vapor for 30 min. Samples were observed using a Tecnai G2 Spirit (FEI Company Hillsboro, Hillsboro, OR, USA) operating at 120 kV transmission electron microscope, equipped with a TVIPS TemCam F-216 CMOS (TVIPS GmbH, Gilching, Germany) Camera for image acquisition.

### 2.11. Data Analysis

Data analysis was performed using GraphPad Prism Version 8.4.2 (GraphPad Software) and Microsoft Excel 2019. Values of * *p* ≤ 0.05 and ** *p* ≤ 0.01 were conventionally considered statistically significant.

## 3. Results

### 3.1. Identification of Lemon Essential Oil Composition

**Figure 2:**
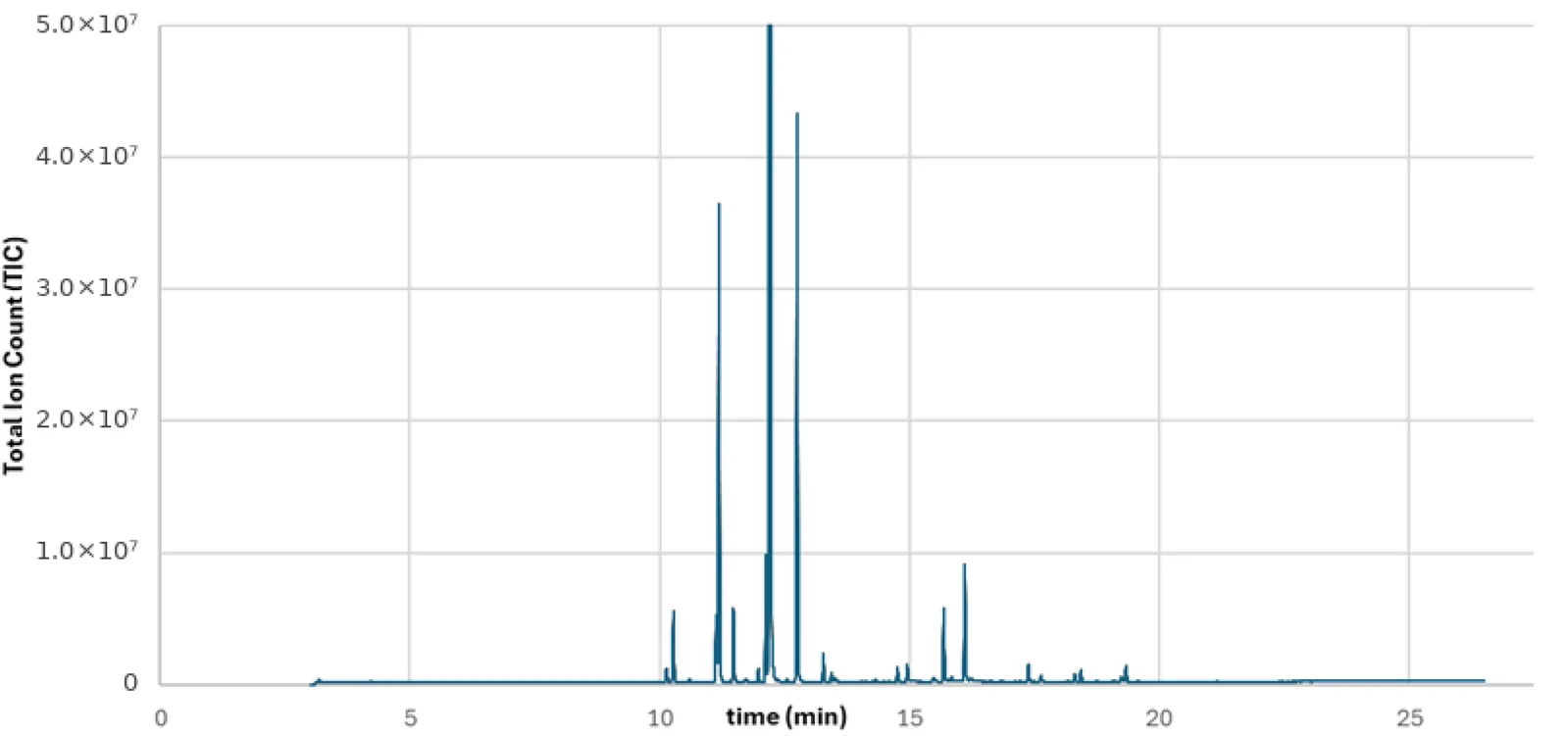
Chromatographic separation of the LEO. Note: the scale is cropped at 5 × 10^7^ to show minor components.

LEO was characterized by means of GC-MS using an Agilent 8890 GC (Agilent, Santa Clara, CA, USA) system coupled to an Agilent 7000E GC/TQ (Agilent, Santa Clara, CA, USA) mass system. The representative chromatogram is reported in Figure 2; meanwhile, the list of the identified compounds is shown in Table 1, together with the estimated composition of the extract on the basis of the relative percentage area. The identification of the individual volatile compounds was performed by combining the information obtained from their mass spectra with their chromatographic retention behavior. In particular, the calculated retention index (RI) of each detected compound was compared with the corresponding literature RI values, providing an additional criterion for compound identification and supporting the assignment based on mass spectral data. To evaluate the reproducibility of the analytical procedure, three independent aliquots of the LEO extract were analyzed under the same experimental conditions. The results obtained from the triplicate analyses were used to estimate the repeatability of the method in terms of relative standard deviation (RSD) for the relative peak areas of the identified compounds. The RSD values were consistently below 5% for both major (e.g., D-limonene RSD = 4.01%) and minor constituents (e.g., p-cymene RSD = 2.23%), indicating good reproducibility of the analytical procedure. The chemical profile was dominated by monoterpene hydrocarbons, with D-limonene representing the major constituent, followed by γ-terpinene, β-pinene, and citral. The relative abundance of these compounds is consistent with the characteristic chemical composition widely reported for LEO [25]. In addition to the major constituents, other monoterpene hydrocarbons, such as α-pinene and p-cymene, were detected in lower amounts, together with a limited number of sesquiterpenes, including bergamotene, which were present at low or trace levels. The chromatographic profile obtained in the present study is therefore consistent with the typical composition of LEO, confirming the predominance of monoterpenes and the characteristic contribution of oxygenated terpenic compounds to the volatile fraction.

### 3.2. Cell Viability at Different Compound Dilutions

**Figure 3:**
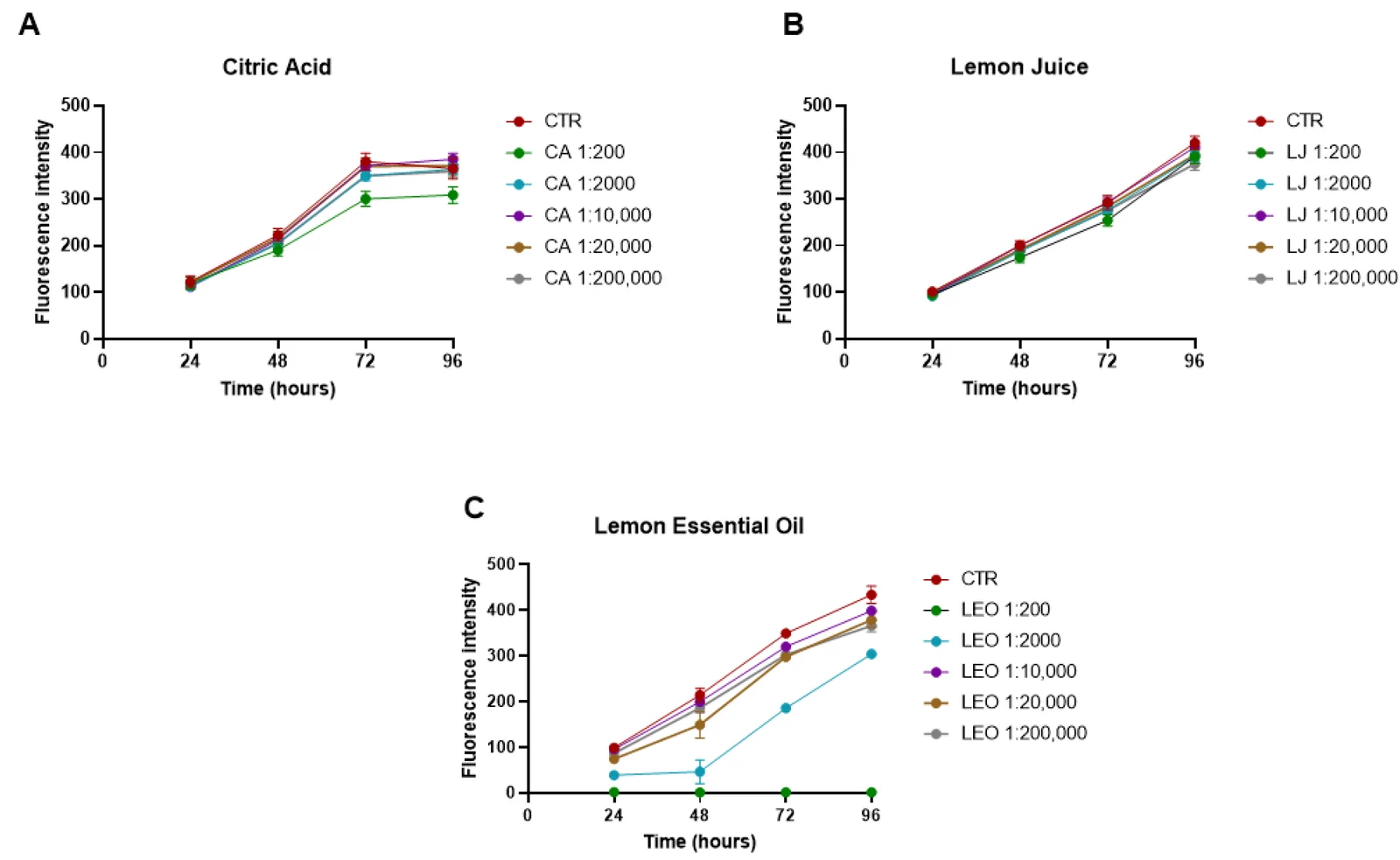
MDCK cell viability with different compound dilutions. Cell viability assessed with fluorescein diacetate assay for the tested compounds: Citric Acid (**A**), Lemon Juice (**B**) and Lemon Essential Oil (**C**); cell viability expressed as fluorescence intensity. Means ± SEM are presented. (n = 3).

Firstly, we aimed to identify the appropriate working dilutions for CA, LJ and LEO that were nontoxic to MDCK cells. Cell viability at different time points and dilutions was assessed using the fluorescein diacetate assay. As shown in Figure 3, serial dilutions of each compound were prepared, spanning up to 1:200,000. Based on the results, 1:2000 was selected as the working concentration for both CA (Figure 3A) and LJ (Figure 3B), as it did not negatively affect cell survival at any time point. For LEO, the 1:10,000 dilution (Figure 3C) was determined to be non-cytotoxic. Importantly, because the FDA assay measures enzymatic activity at a specific time point rather than cumulatively, the overall increase in fluorescence intensity observed from 24 h to 96 h reflects the active proliferation and increasing number of viable MDCK cells in the wells over time.

**Figure 4:**
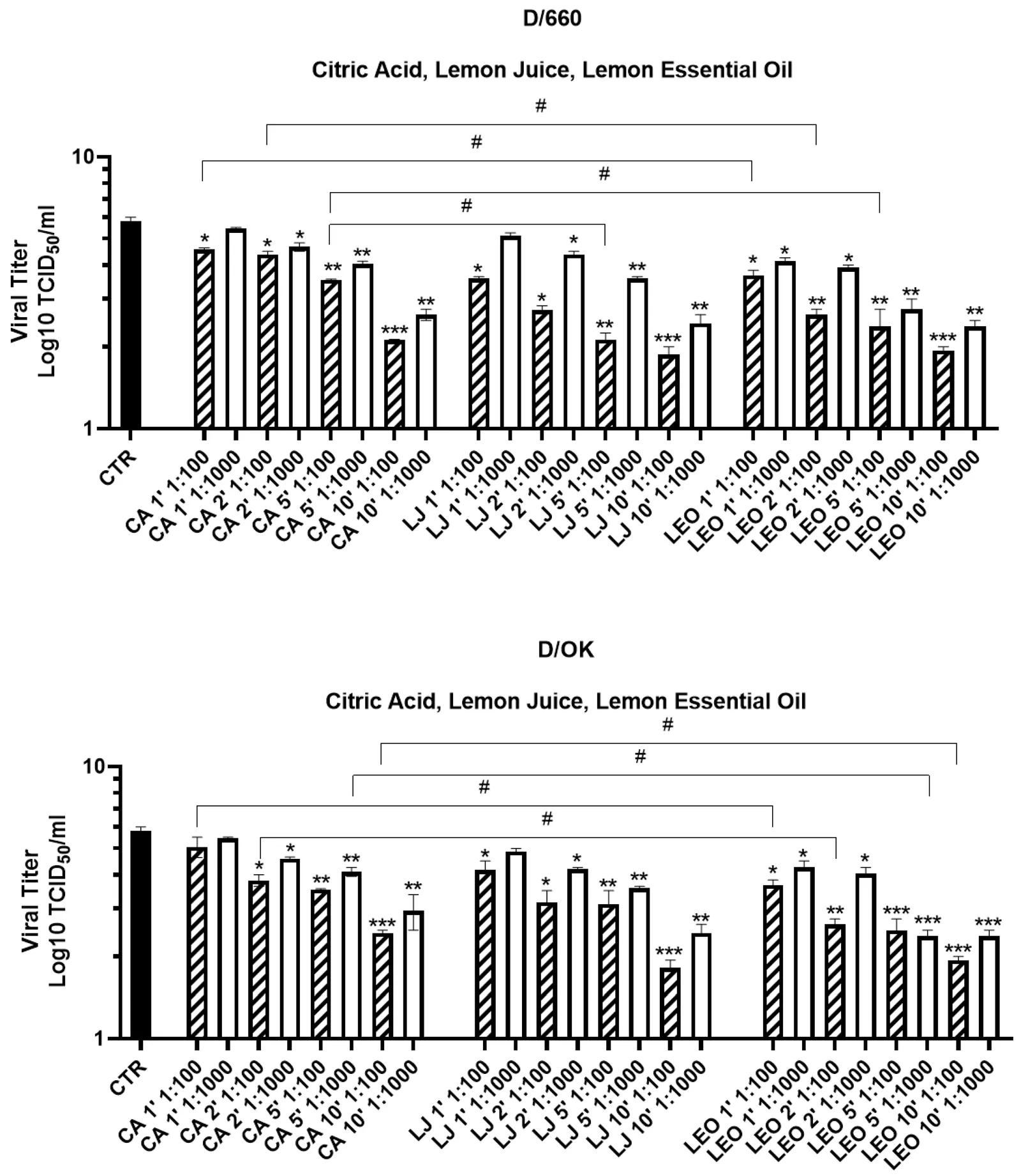
IDV titration in MDCK cells after CA, LJ and LEO treatment at different time points and two different concentrations. Viral infectivity is expressed as log_10_ TCID_50_/mL. (n = 3; # *p* ≤ 0.05, * *p* ≤ 0.05, ** *p* ≤ 0.01, *** *p* ≤ 0.001 indicates statistically significant difference). Statistical significance was determined by comparing each treated sample with the corresponding untreated virus control (indicated with *) or comparing treated samples with each other (indicated with #). Means ± SEM are presented. CTR = control, CA = Citric Acid, LJ = Lemon Juice, LEO = Lemon Essential Oil. For CA, 1:100 dilution corresponds to 2.4 mM and 1:1000 to 0.24 mM.

In addition, higher concentrations above the cytotoxic threshold (1:100 and 1:1000) were included in the virucidal assays to evaluate the potential rapid virucidal effects, as shown in the subsequent results and illustrated in Figure 4.

### 3.3. Citric Acid, Lemon Juice and Lemon Essential Oil Reduce Viral Titers at High Concentration

Initially, the virucidal effects of CA, LJ, and LEO against both D/660 and D/OK were assessed following exposure of the viruses to high concentrations of the compounds (above the cytotoxic threshold). As shown in Figure 4, treatment with two concentrations of each compound (1:100 and 1:1000) resulted in a significant concentration-dependent reduction in viral titers across all time points. For both IDVs, CA produced limited but statistically significant antiviral effects, with the greatest reduction observed after 10 min of exposure at the 1:100 dilution and at the 1:1000 dilution. LJ exhibited stronger inhibitory activity than CA, with a notable decrease in titers (approximately 1.5–2 log_10_ units relative to control, which was 5.8 log_10_) even after 1 min of exposure at the 1:100 dilution, suggesting virus-inhibitory effects, while CA exposure was capable of decreasing the viral titer by 0.6 log10 TCID_50_/mL at 1 min exposure.

Similarly, LEO exhibited pronounced and rapid virucidal activity. At the 1:100 dilution, LEO reduced the infectious titers of both IDV lineages by 2.5 log_10_ units (a 99.96% reduction; *p* ≤ 0.01) after just 1 min of exposure, with the reduction reaching 4 log_10_ units (a 99.99% decrease) at 10 min. Even at the higher dilution of 1:1000, LEO maintained strong efficacy, decreasing viral titers by 2.0 log_10_ units at 1 min and up to 2.8 log_10_ units at 10 min (*p* ≤ 0.01).

Overall, these results indicate a dose-dependent antimicrobial effect for all tested lemon-derived products, with LEO exhibiting the greatest efficacy. Notably, significant reductions in viral titer were observed at higher concentrations and were evident at the earliest time points (e.g., 1 min), highlighting the rapid virucidal action of these compounds. These findings suggest that lemon-derived compounds can exert measurable virus-inhibitory effects when applied at sufficiently high concentrations, particularly with short exposure periods.

### 3.4. Lemon Juice and Lemon Essential Oil Reduce Viral Titers at Non-Cytotoxic Concentrations

**Figure 5:**
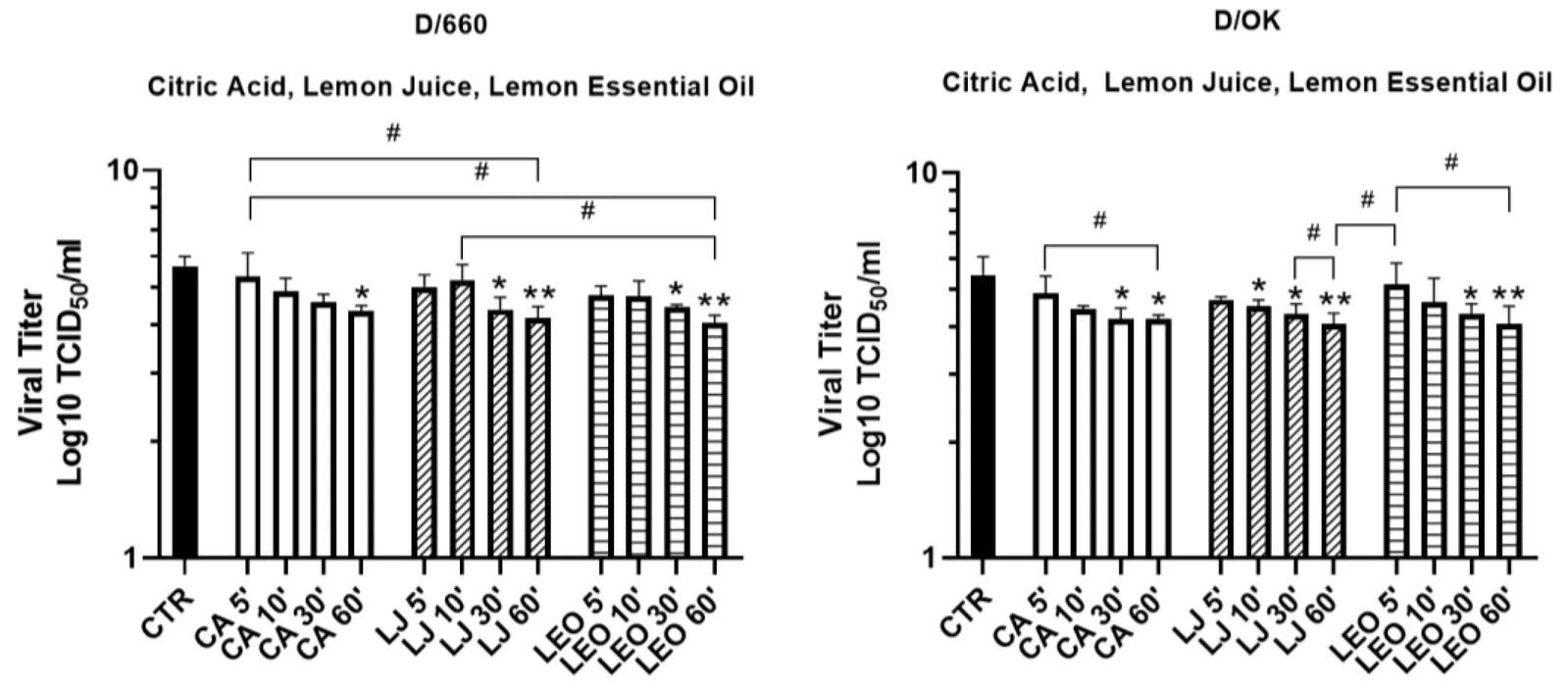
IDV titration in MDCK cells after CA, LJ and LEO treatment at different time points. Virucidal activity of CA (1:2000 dilution), LJ (1:2000 dilution) and LEO (1:10,000 dilution against IDV. Viral infectivity is expressed as log_10_ TCID_50_/mL. Asterisks (*) indicate statistically significant differences between the indicated treated sample and the corresponding untreated virus control. Hash symbols (#) indicate statistically significant differences between the indicated treated conditions. Only statistically significant comparisons are annotated (n = 3; # *p* ≤ 0.05, * *p* ≤ 0.05, ** *p* ≤ 0.01 indicate statistically significant differences). Means ± SEM are presented. CTR = control, CA = Citric Acid, LJ = Lemon Juice, LEO = Lemon Oil. For CA, 1:2000 dilution corresponds to 0.12 mM.

D/660 and D/OK were exposed to the compounds for varying time points to evaluate the virucidal effect of LJ and LEO at previously determined non-cytotoxic dilutions (1:2000 for LJ and 1:10,000 for LEO). As shown in Figure 5, at the non-cytotoxic concentration of 0.12 mM (1:2000 dilution CA showed a time-dependent effect on IDV infectivity. For D/660, CA produced a limited reduction at the shorter exposure times, while a statistically significant reduction compared with the untreated virus control was observed only after 60. In contrast, both LJ and LEO produced a significant reduction in viral titer over time. Compared with the untreated control, samples incubated with LJ and LEO for 5 or 10 min showed modest decreases in TCID_50_ values, although these differences were not statistically significant. Longer exposures of 30 and 60 min led to a more pronounced reduction, with log_10_ TCID_50_/mL decreasing from 5.5 to 4.25 for LJ (60 min) and to 4 for LEO (60 min). Similarly, for D/OK, all compounds reduced viral titers. In particular, treatment with CA, LJ, and LEO for 30 and 60 min produced a statistically significant decrease. Specifically, log_10_ TCID_50_/mL decreased from 5.5 to log_10_ TCID_50_/mL 4.0 following 60 min exposure to LJ and LEO. Additionally, a significant reduction was observed at 10 min for LJ.

These findings indicate that prolonged exposure to LJ and LEO enhances their virus-inhibitory effect, suggesting a time-dependent inactivation of viral particles.

### 3.5. Immunofluorescence Analysis Highlights Reduced Influenza D Virus Nucleoprotein After Lemon Juice and Lemon Essential Oil Treatment

**Figure 6:**
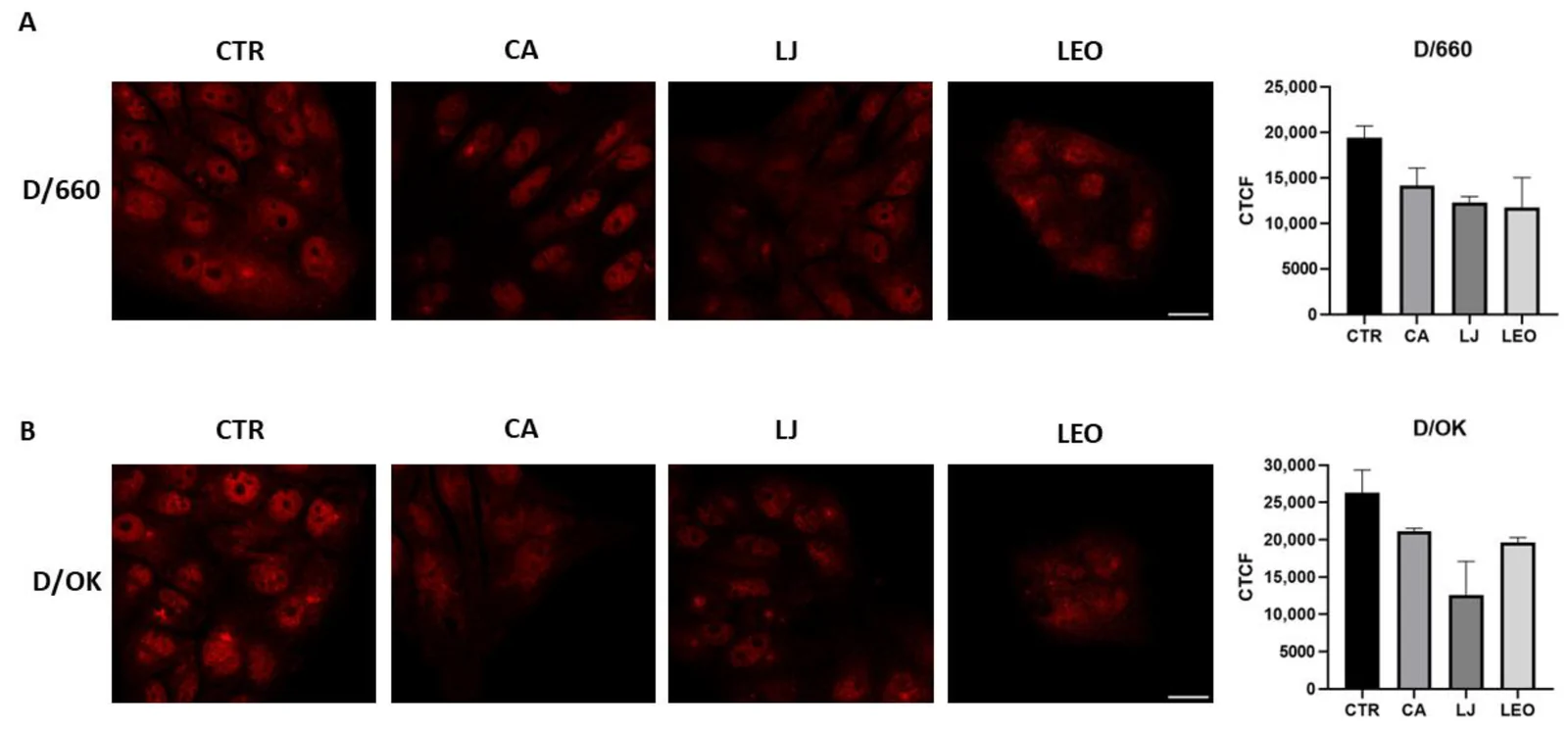
IDV NP fluorescence following exposure of viral suspensions to the tested compounds. IDV NP immunofluorescence analysis after treatment with CA, LJ, and LEO against D/660 (**A**) and D/OK (**B**). Means ± SEM are presented. Representative images (medial optical sections) are shown (scale bar 50 μm). n = 2; ≥25 cells from different experiments; for each experiment, the mean CTCF value was calculated from the analyzed cells, and the two independent experiments were subsequently considered as experimental replicates. Data are presented descriptively without statistical significance testing. CTR = control, CA = Citric Acid, LJ = Lemon Juice, LEO = Lemon Oil. CTCF = Corrected Total Cell Fluorescence.

To assess the effect of the compounds on IDV NP expression, D/OK and D/660 viruses were pre-treated with CA, LJ and LEO for 60 min and then used to infect MDCK cells. After 96 h, cells were fixed and stained for IDV NP. As shown in Figure 6, all compounds reduced NP signal in both D/660 (Figure 6A) and D/OK (Figure 6B). LEO had the greatest effect on NP expression in D/660, while LJ was most effective in D/OK. Notably, NP reduction following LJ treatment was higher than that observed with CA, suggesting that components present in LJ, in addition to its acidic pH, may contribute to the inhibition of NP expression.

The reduced NP signal indicates a lower level of IDV infection following exposure of the viral suspensions to the tested compounds. However, because the compounds were not removed from the virus preparations before inoculation of the cells, the present experimental design does not allow the reduction in NP signal to be attributed exclusively to the initial virucidal effect. Potential effects of residual compounds on cell susceptibility or subsequent stages of infection cannot be completely excluded.

### 3.6. Transmission Electron Microscopy Reveals Morphological Alterations of IDV Particles Following Treatment with Citric Acid, Lemon Juice and Lemon Essential Oil

To evaluate the impact of the compounds on IDV structure, D/660 and D/OK viruses were exposed to CA, LJ, or LEO for 60 min, and samples were prepared for negative staining and observation by transmission electron microscopy.

**Figure 7:**
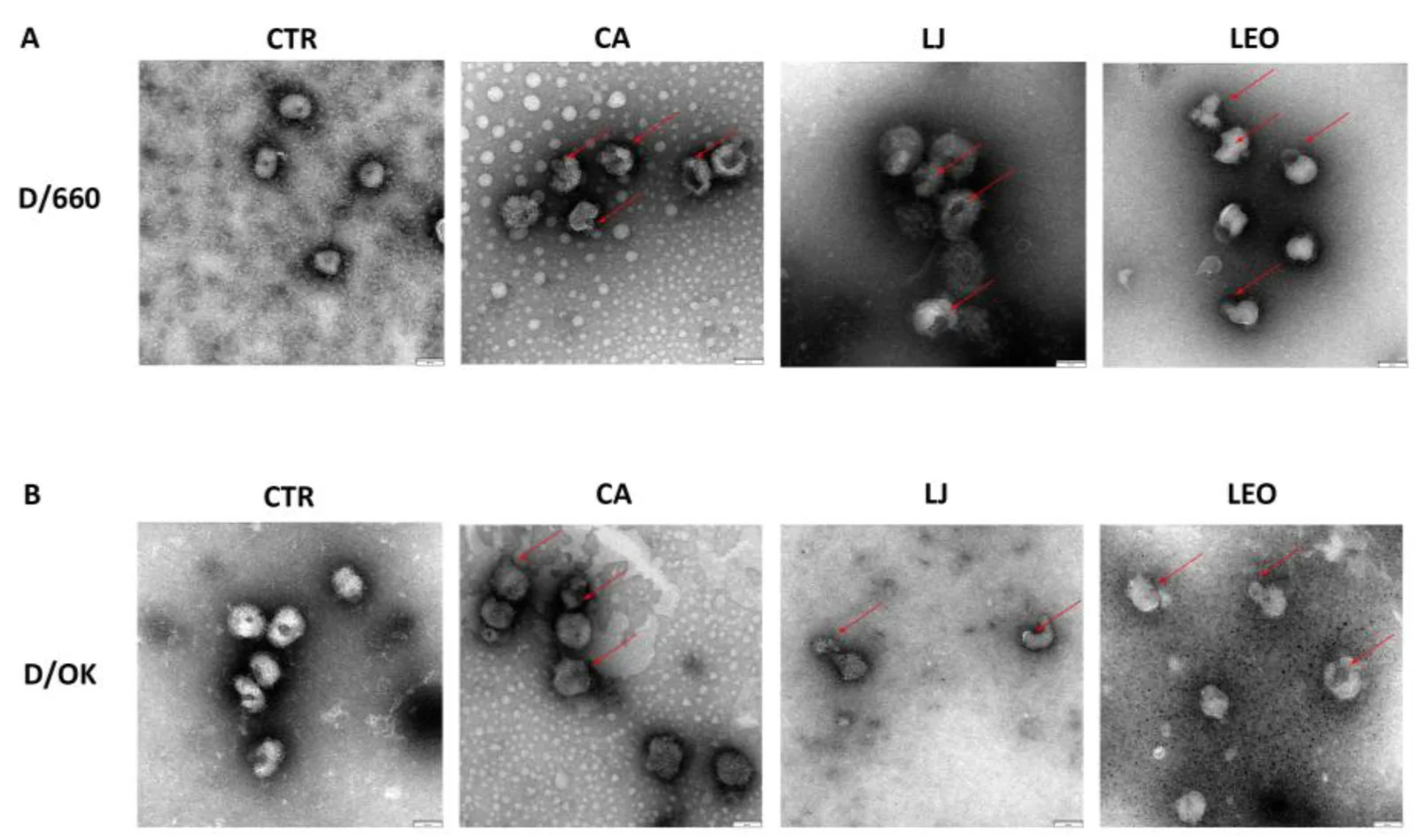
Transmission electron microscopy (TEM) of IDV particles following treatment with CA, LJ and LEO. Virus suspensions were incubated with CA, LJ (1:2000 dilution) or LEO (1:10,000 dilution) for 1 h. Untreated virus served as the control. Negative staining was performed. Representative images are shown. Arrows indicate representative viral particles displaying morphological alterations compared with the untreated control. CTR = control, CA = Citric Acid, LJ = Lemon Juice, LEO = Lemon Oil. Scale bars = 80 nm.

As shown in Figure 7, untreated control samples displayed predominantly intact viral particles with the expected overall morphology (typical well-defined spherical structures), although a small proportion of particles showing morphological alterations was also observed. In contrast, compound-treated samples showed a higher frequency of particles exhibiting morphological alterations, including irregular particle morphology and apparent alterations in the overall organization of the viral particles and loss of surface integrity.

IDV altered particles following treatment with the tested compounds are indicated by arrows in Figure 7. In addition, we quantified the damaged viral particles in each condition and reported the percentage of altered/damaged viral particles in Table 2. Indeed, for the descriptive assessment, at least 50–60 viral particles were evaluated for each experimental condition. The proportion of particles displaying morphological alterations was higher in treated samples than in the corresponding controls. For D/660, 68%, 76%, and 80% of the particles examined following CA, LJ, and LEO treatment, respectively, compared with 17% in the untreated control, were altered. For D/OK, the corresponding values were 54%, 81%, and 86%, compared with 10% in the untreated control.

Overall, all three treatments were associated with morphological alterations compared with the untreated controls, with LJ and LEO showing a higher proportion of altered particles under the experimental conditions tested. These observations provide supportive morphological evidence of an effect of the tested compounds on IDV particle integrity and establish a specific mechanism of virion disruption.

## 4. Discussion

Natural products have long served as an important source of therapeutic agents, with approximately 50% of current drugs derived from compounds of natural origin. Their continued relevance is driven by urgent therapeutic needs, the structural and biological diversity of natural metabolites, and advances in analytical and production techniques that facilitate their identification and development [27].

In this study, we demonstrate that LJ and LEO exhibit significant, time-dependent antiviral activity against both IDV D/660 and D/OK. High concentrations of these compounds were tested to evaluate the virucidal activity over short exposure times, highlighting their potential use for surface disinfection. Additionally, their virucidal potential at non- cytotoxic concentrations was also investigated as a possible treatment approach.

We further show that LJ and LEO reduce viral NP expression in infected MDCK cells and induce structural damage to IDV. Together, these findings support the occurrence of direct virion inactivation under the experimental conditions tested. The morphological alterations observed by TEM are consistent with an effect of the tested compounds on virion integrity; however, these observations should be considered supportive morphological evidence and do not by themselves establish a specific physically disruptive mechanism.

In this study, we demonstrate that LJ and LEO exhibit significant, time-dependent antiviral activity against both IDV D/660 and D/OK. Specifically, at high concentrations above the cytotoxic threshold (1:100 dilution), a rapid and significant reduction of approximately 1.5–2 log10 units was observed within just 1 to 2 min of exposure. Furthermore, at the maximum non-cytotoxic working concentrations (1:2000 dilution for LJ and 1:10,000 dilution for LEO), a pronounced time-dependent reduction was observed after prolonged exposure (30 and 60 min), reducing viral titers from 5.5 to 4.0 log10 TCID50/mL. In addition, although CA contributed to the observed reduction in viral infectivity, the greater activity observed for LJ under the experimental conditions tested suggests that additional naturally occurring constituents may also contribute to its antiviral effect. However, because differences in buffering capacity were not specifically investigated, the relative contribution of acidity and non-acid phytochemicals cannot be conclusively determined from the present study. Despite this limitation, our findings suggest that virion inactivation is dependent on the duration of compound–virus interaction, consistent with a kinetic virucidal mechanism. These results are consistent with previous studies showing the rapid virucidal activity of citrus-derived products. Lanave et al. demonstrated that undiluted LJ reduced feline calicivirus titers by approximately 4.5 log_10_ TCID_50_ within 30 s, achieving complete inactivation within 1 min. Similarly, Pellegrini et al. reported time-dependent virucidal activity of LEO against feline calicivirus, with extended exposure (up to 8 h) further increasing viral inactivation. Although feline calicivirus is a non-enveloped norovirus surrogate, the rapid loss of infectivity upon citrus treatment supports the notion that lemon-derived phytochemicals can exert strong physicochemical stress on viral particles. The significant virucidal effects here for IDVs after 30–60 min likely reflect similar mechanisms, potentially modulated by the presence of the viral envelope. Indeed, enveloped viruses may be more susceptible to membrane-active compounds present in LJ and EO, including limonene, citral, γ-terpinene, and β-pinene.

Although CA exhibited antiviral activity against IDV, it did not fully reproduce the extent of viral titer reduction observed in LJ and LEO under the experimental conditions employed. This observation suggests that the antiviral activity of lemon-derived products is likely not attributable solely to CA but may also involve the contribution of other bioactive constituents present in these matrices.

This observation aligns with findings of Yu et al., who demonstrated that IDV was resistant to inactivation by low pH; after treatment at pH 3.0 for 30 min, the virus lost only approximately 20% of its original infectivity [18]. Notably, LJ contains flavonoids, phenolic acids, and organic acids, which may play a pivotal role in virucidal and antiviral activity. On the other hand, LEO is mainly composed of monoterpenes, with limonene typically constituting up to ~70% of the oil, although its concentration varies depending on extraction method and cultivar [21]. The enhanced antiviral activity of LJ and LEO relative to CA alone likely reflects a synergistic action of acidic pH and volatile phytochemicals capable of penetrating and destabilizing viral structures. However, future pivotal in vitro studies should focus on the isolation and quantitative characterization of these specific compound classes, evaluating potential variations due to cultivar, seasonality, and geographical origin. This approach will enable better standardization of the extracts and allow assessment of synergistic antimicrobial effects and cytotoxicity levels.

Multiple studies support the antiviral properties of these compounds: EOs from *Lippia* spp., *Rosmarinus officinalis*, and *Eucalyptus globulus* have demonstrated virucidal activity against avian influenza virus H7N3 [28]. In addition, citrus EOs and their monoterpenes have been reported to exhibit antiviral activity against herpes simplex virus, adenovirus, and other respiratory viruses [29].

IDV pre-treated with LJ or LEO resulted in a significantly reduced NP immunofluorescent signal in infected MDCK cells at 96 h post-infection. The extent of NP reduction showed lineage-dependent trends, with LEO producing the greatest reduction observed for D/660 and LJ producing the greatest reduction observed for D/OK within their respective experimental comparisons. These findings indicate that exposure of IDV to the tested compounds was associated with a reduced level of subsequent infection, consistent with the reduction in infectious viral titers observed in the TCID_50_ assays (at 96 h end-time experiment). However, because the compounds were not removed from the virus preparations before inoculation, the reduced NP signal cannot be attributed exclusively to direct virion inactivation. Residual compounds may have remained present during the infection period and could potentially affect cell susceptibility or subsequent stages of infection. Therefore, the immunofluorescence results should be interpreted as supportive evidence of reduced infection following exposure of the virus to the tested compounds, rather than as evidence of a specific antiviral mechanism.

The observed differences in NP reduction between D/OK and D/660 may reflect lineage-specific variations in viral envelope composition, replication kinetics, or susceptibility to the acidic and bioactive components of LJ and LEO, highlighting the importance of evaluating multiple strains when assessing antiviral efficacy. This reduction in NP expression is consistent with the significant decrease in infectious particles measured by TCID_50_, indicating that fewer virions remained capable of initiating productive infection. Indeed, the immunofluorescence signal acquired after 96 h reflects the cumulative outcome of the initial viral inactivation followed by multi-cycle viral spread, reinforcing the data obtained within the virucidal assays. This highlights that the antiviral effects exerted by the tested lemon compounds can reduce viral antigen production and reduce cell-to-cell propagation over an extended culture period.

It is also possible that certain compounds interfere with early stages of the viral life cycle after entry, potentially affecting genome release, transcription, or early replication events. Furthermore, lemon-derived compounds may activate cellular molecular pathways that can act as a protection against viral infection. Similar reductions in viral protein expression following direct virucidal treatments have been reported in studies investigating EOs against herpes simplex virus-1 and influenza viruses [29]. The immunofluorescence findings should be interpreted as complementary to the TCID_50_ measurements. The reduction in NP-associated fluorescence was consistent with the lower infectious titers observed after compound exposure, providing a supportive cellular readout of reduced infection.

Moreover, negative-stain TEM revealed alterations in IDV morphology following exposure to CA, LJ, or LEO. Approximately 50–60 viral particles were evaluated for each experimental condition, and a higher proportion of morphologically altered particles was observed in treated preparations compared with untreated controls. Such morphological alterations are consistent with a possible effect of the tested compounds (CA, LJ, and LEO) on viral particle morphology and integrity. Citrus EOs are known to interact with lipid bilayers, increasing membrane fluidity, altering permeability, and ultimately inducing membrane disruption [30]. However, given the descriptive nature of the TEM assessment, these findings should be considered supportive rather than definitive evidence of direct virion disruption.

However, this study has some limitations. The in vitro design necessitates further investigation in more complex biological systems that account for host immune responses and mucosal barriers. Although MDCK cells provide a robust model for preliminary screening, they may not fully replicate the physiological conditions of the natural target tissue, and in vivo studies will be essential to assess efficacy, safety and therapeutic outcomes. Indeed, the experiments were performed using the MDCK cell line, a well-established and widely used model for influenza virus propagation, titration, and antiviral evaluation; however, this system does not fully reproduce the complexity of the natural respiratory environment of susceptible host species. Validation in primary respiratory epithelial cells derived from natural hosts, such as bovine or porcine cells, would provide further insight into the biological relevance and potential applicability of the observed antiviral effects. Future studies should therefore include host-specific cellular models to confirm these findings under conditions that better reflect natural infection situations.

An additional limitation of the present study is that the tested compounds were not removed before viral inoculation of cells in the immunofluorescence experiment. Therefore, although the observed reduction in viral replication is compatible with a direct antiviral/virucidal effect, a contribution of compound-mediated modulation of host cell susceptibility cannot be completely excluded. In addition, although serial dilution substantially reduces the concentration of LEO carried over into the titration assay, a residual contribution of LEO-mediated virucidal activity during the titration procedure cannot be completely excluded. Similarly, potential interference of residual LEO with MDCK cells cannot be definitively excluded.

Moreover, the chemical characterization was not carried out for LJ since the aim of this study was to evaluate the antiviral activity of the whole LJ; on the other hand, we focused our attention on characterizing LEO composition even though the individual compounds present in this compound were not tested separately yet. Finally, higher-resolution structural analyses, such as cryo-electron microscopy, could provide deeper insight into the molecular interactions between lemon-derived compounds and viral particles.

In conclusion, our findings demonstrate that Citrus limon-derived products, particularly LJ and LEO, exhibit potent and rapid antiviral activity against IDV lineages D/660 and D/OK. This effect is consistent with direct virion inactivation, while the morphological alterations observed by transmission electron microscopy provide supportive evidence of changes in viral particle integrity. However, the precise mechanism underlying these effects cannot be established from the present study. Furthermore, this initial viral inactivation successfully translates into a sustained reduction in multi-cycle viral propagation and viral protein accumulation in host cells over time. Overall, these natural compounds represent promising candidates for further investigation in the development of novel antiviral strategies against emerging zoonotic orthomyxoviruses.

Considering that IDV has been detected across diverse animal species, in humans, and in diverse environments, including hospital emergency rooms, airports, and the human–pig interface, these compounds may have utility beyond direct virucidal treatment.

The virucidal activity observed in vitro warrants further investigation of Citrus limon-derived products as candidate sources of anti-IDV activity. Potential applications include surface disinfection and preventive formulations such as hand sanitizers or nasal sprays. Translation of these findings into practical applications would require dedicated studies addressing formulation, stability, safety, effective exposure conditions, and efficacy in relevant natural-host cell models and, ultimately, appropriate in vivo systems.

Additionally, they may serve as adjunctive agents in livestock management practices. Further studies are warranted to better elucidate their mechanisms of action and to evaluate their effectiveness and feasibility in real-world antiviral and sanitization strategies, particularly those targeting respiratory viruses, such as IDV.

## 5. Conclusions

In conclusion, our findings demonstrate that Citrus limon-derived products, particularly LJ and LEO, exhibit virucidal activity against the IDV lineages D/660 and D/OK under the in vitro conditions investigated. These findings provide a basis for further investigation of Citrus limon-derived compounds as potential sources of antiviral activity against IDV, including potential surface disinfection and topical formulations. However, the present results do not establish their suitability for therapeutic, topical, environmental, aerosol, or livestock applications. Further studies are required to determine formulation stability, safety, effective concentrations and exposure conditions, and activity in relevant natural-host cell models before any practical application can be considered.

## Figures and Tables

**Table 1:** Identified compounds and corresponding retention time (min), percentage composition on the basis of the peak area, and percentage area with respect to the most intense peak (D-Limonene).

Compound	Retention Time (min)	Calculated RI	Library RI	Area %	Area % Max
α-Thujene	10.13	929	928	0.29	0.43
α-Pinene	10.27	936	937	1.40	2.10
Sabinene	11.13	976	974	1.22	1.82
β-Pinene	11.18	979	979	9.25	13.86
β-Myrcene	11.48	993	991	1.32	1.98
α-Terpinene	11.99	1019	1018	0.26	0.38
*p*-Cymene	12.12	1028	1025	2.36	3.54
D-Limonene	12.21	1033	1031	66.7	100.00
γ-Terpinene	12.75	1063	1060	10.5	15.80
α-Terpinolene	13.27	1092	1080	0.53	0.79
Linalool	13.44	1101	1099	0.20	0.29
Terpinen-4-ol	14.76	1184	1177	0.30	0.45
γ-Terpineol	14.96	1196	1187	0.32	0.48
Neral	15.69	1246	1240	1.30	1.94
Citral	16.11	1274	1273	2.12	3.17
Nerol Acetate	17.38	1366	1362	0.31	0.46
α-Guaiene	18.30	1437	1437	0.14	0.21
Bergamotene	18.43	1446	1435	0.23	0.38
δ-Selinene	19.24	1502	1485	0.10	0.18
γ-Bisabolene	19.34	1518	1515	0.30	0.45
Total identified Components				99.15	

**Table 2:** Altered viral particles counted in each experimental condition in the acquired TEM images. At least 50–60 viral particles were counted in each experimental condition; a percentage of damaged particles is reported to simplify the table readout.

Virus	Treatment	Total Particles Analyzed	Altered Particles	Altered (%)
D/660	Control	58	10	17
D/660	CA	50	34	68
D/660	LJ	51	39	76
D/660	LEO	54	43	80
D/OK	Control	62	6	10
D/OK	CA	61	33	53
D/OK	LJ	58	47	81
D/OK	LEO	63	54	86

## Data Availability

The original contributions presented in the study are included in the article; further inquiries can be directed to the corresponding author.
